# P14 Optimization of real-time sequencing for rapid detection of bacterial species and antimicrobial resistance in blood and urine infections

**DOI:** 10.1093/jacamr/dlad077.018

**Published:** 2023-08-02

**Authors:** Maria Getino, Rebecca Stout, Frances Davies, Elita Jauneikaite

**Affiliations:** Imperial College London, London, UK; Imperial College London, London, UK; Imperial College Healthcare NHS Trust, London, UK; Imperial College London, London, UK; Imperial College Healthcare NHS Trust, London, UK; Imperial College London, London, UK

## Abstract

**Objectives:**

To optimize the process to obtain bacterial DNA directly from human urine and blood samples to produce high-quality genomic data using long-read Nanopore sequencer.

**Methods:**

A total of 22 blood cultures and 24 urine samples positive for *Escherichia coli* or *Klebsiella pneumoniae* and corresponding isolates were collected from the microbiology diagnostics laboratory. DNA isolation optimization was performed testing three different sample volumes and seven DNA extraction kits. DNA concentration was quantified with Qubit fluorometer. Nanopore sequencing was performed on a MinION Mk1C device in R9.4.1 flow cells using the Rapid Barcoding Kit. Bacterial species and antimicrobial resistance (AMR) genes were determined with EPI2ME ARMA pipeline. Genomes were assembled with Flye, polished with Medaka and remaining human contigs removed with Krakentools. Bacterial isolates from the same samples were also sequenced using Illumina technology. Illumina and Nanopore assemblies were quality checked with Quast, Kraken was used to identify bacterial species, MLST for multilocus sequence typing and ABRicate with ResFinder database to detect AMR genes.

**Results:**

A total of 72 DNA extractions from 24 urine and 22 blood samples with different kits were performed to find the optimal extraction protocol for each sample type. DNA from urine samples was isolated from 5 mL, while 1 mL was best for blood cultures. We found that the host depletion step prior to DNA extraction using MolYsis basic 5 Kit (VH-Bio) was essential. DNA from urine samples was extracted with GenElute Bacterial Genomic DNA Kit (Merck) and QIAamp Biostic bacteremia Kit (Qiagen) was best to allow successful bacterial genome sequencing for blood samples. Bacterial isolates from 11 urine and 1 blood sample (out of 54 sequenced) were successfully sequenced. In all 12 cases, the highest percentage of bacterial reads matched with the causative organism found during routine microbiology diagnostics (*E. coli*). A variety of clinically relevant AMR genes were detected, such as *dfrA* and *bla*_CTX-M_ genes, which matched the phenotypic antimicrobial susceptibility testing results. Comparison of genomic assemblies obtained through Nanopore sequencing of 10 urine samples and Illumina sequencing of the corresponding clinical isolates showed a high degree of matching, validating Nanopore sequencing as an alternative option to support diagnosis and treatment choice.

**Conclusions:**

We have successfully optimized the protocols to extract and sequence pathogenic bacterial DNA directly from blood and urine samples. Short-read sequencing confirmed results acquired by Nanopore sequencer, endorsing long-read sequencing in clinical settings to support timely diagnosis and treatment.

